# Transcriptional regulation of autophagy‐lysosomal pathway in cancer

**DOI:** 10.1111/1759-7714.13287

**Published:** 2020-01-08

**Authors:** Xinzhong Chang, Ruihua Dong

**Affiliations:** ^1^ The First Surgical Department of Breast Cancer Cancer Institute and Hospital, Tianjin Medical University Tianjin China; ^2^ Oncology department Weifang People's Hospital Weifang China

**Keywords:** Autophagy, lysosome, oncoprotein, oncosuppressor, transcriptional regulator

## Abstract

The transcriptional regulation of autophagy‐lysosomal pathway adapts to cellular stress and enables advanced cancer cells survive. This pathway plays an oncopromoting or oncosuppressing role, depending on context‐dependent stresses and treatment resistance. It remains controversial whether this pathway represents a target for drugs, although autophagy‐lysosomal inducers and inhibitors have been tested in clinical trials for cancer treatment. Therefore, identifying the transcriptional regulators of autophagy‐lysosomal pathway may lead to the development of effective cancer treatment and the improvement of the existing targeted cancer therapies. In this review, we summarize findings from several published studies on transcriptional regulation of autophagy‐lysosomal pathway in cancer biology, and evaluate its functional role as a therapeutic target.

## Introduction

The metabolic reprogramming maintains the survival, proliferation, and metastasis of cancer cells in adverse microenvironmental conditions. Surgery, chemotherapy, radiation, hormone therapy, biological therapy, and targeted therapies are often applied as cancer treatment strategies. In some cases, viable nonproliferating tumor cells remain quiescent for years, eventually to chemotherapy becoming resistant, resulting in rapid disease progression and treatment failure.^1^ More investigations of alternative treatment of cancer are demanded for better clinical outcomes. The manipulation of autophagy and its regulatory pathway has becoming an emerging anticancer strategy.

Three forms of autophagy can be distinguished morphologically: macroautophagy, microautophagy and chaperone‐mediated autophagy (CMA).^2^ Here, we focus on macroautophagy (autophagy). The autophagy‐lysosomal function is a highly context‐dependent and spatiotemporally dynamic process, critical for cellular homeostasis and cell remodeling. Cellular components are sequestered into double‐membrane vesicles and delivered to the lysosome for degradation and for recycling to other intracellular compartments. This pathway plays an important role in intracellular biomolecular degradation and recycling. During autophagy, aggregated and misfolded proteins and damaged organelles are delivered to the lysosome in double‐membrane vesicles called the autophagosomes, which then fuse with lysosomes and form single‐membrane vesicles called autolysosomes.

Autophagy and lysosomal activities are critical for normal cellular function and are coordinately regulated under stressful conditions to ensure efficient clearance and recycling of damaged proteins and organelles. Under normal physiological situations, basal level autophagy maintains homeostasis. Under stressful conditions, autophagy can be upregulated in response to pathogenic, metabolic, nutritional, genotoxic, oxidative and proteotoxic cues so as to sustain an adaptive response with cytoprotective functions. Therefore, it can sustain the survival and proliferation of tumor cells during microenvironmental stress or systemic therapy to support tumor growth, invasion, and metastasis. As previously reported, in quiescent gastrointestinal stromal tumor (GIST) cells, tyrosine kinase inhibitor imatinib induces autophagy to promote survival. A combination of imatinib treatment and autophagy inhibition efficiently enhanced GIST cytotoxicity to abrogate cellular quiescence and acquired resistance both in vitro and in vivo.^3^

On the other hand, sometimes,autophagy is a barrier against cell‐damaging events, including malignant transformation. Autophagy serves as an oncorepressor and some oncosuppressor proteins can stimulate autophagy while several oncoproteins inhibit autophagy.^4^ Emerging evidence suggests that autophagy‐induced cell death or the inhibition of autophagy may represent novel therapeutic strategies against cancer. Thus, manipulating autophagy may represent an alternative strategy for improving anticancer therapies. Our molecular understanding of autophagy is rapidly evolving and autophagy‐oriented clinical trials have identified more autophagy‐modulating compounds with therapeutic benefit.^5^ The autophagy‐lysosomal process is a genetically programmed process regulated by fine‐tuned interactions between cellular autophagy signaling pathways and autophagy‐lysosomal regulators, including the transcription factors and their coregulators.^6^ This process controls the flux of autophagy and exerts critical functions in cell fate decision. The transcriptional and the epigenetic regulation of the autophagy‐lysosomal function and its signaling pathways in cancer cells, therefore, need to be summarized. In this review, we focus on the transcriptional regulation of autophagy‐lysosomal function and regulation in tumorigenesis.

## Autophagy‐lysosomal function in cancer

Autophagy plays a key role in cancer development, but whether this role is tumor suppressive or tumor promoting remains controversial and depends on the distinct cellular context. It is generally accepted that autophagy suppresses the initiation and development of tumors in the early stages of cancer and promotes tumor survival and growth in advanced stages.^7^ Thus, autophagy is a double‐edged sword that can either facilitate or impede tumorigenesis.

The metabolism of cancer cells is altered to meet the energy demands of survival, proliferation, and metastasis. This increases the speed of energy production by upregulating aerobic glycolysis, but reduces the efficiency of energy production by decreasing electron transport chain activity in the mitochondria. This is called the “Warburg effect.”^8^ Glucose is the main substrate for aerobic glycolysis, therefore, glutamine is mainly used in the mitochondrial TCA cycle and the generation of NAD(P)H and fatty acids. Some tumor cells (including activated Myc) depend on glutaminolysis for survival and upregulate glutamine transporters and enzymes.^9^ Basal autophagy can be promoted by ammonia produced by glutaminolysis, protecting cancer cells from TNFα‐induced apoptosis and limiting proliferation under stressful conditions.^10^ Moreover, autophagy can interact with other pathways such as Ras and p53 in tumor cells to regulate tumorigenesis. Some studies have shown that in the mouse models of oncogenic K‐ras‐induced lung cancer, ATG5 or ATG7 deletion diminished the overall lung tumor burden and abnormal mitochondria accumulated in the tumor cells.^11^

Tumor cells use autophagy to respond to unfolded protein response (UPR). UPR protects cells from alleviated stress caused by the accumulation of misfolded proteins in the endoplasmic reticulum (ER).^12^ UPR can be promoted by multiple stressors in tumor cells, such as activation of the oncogenic transcription factor Myc or hypoxia. Oncogenic activation of c‐Myc promotes translation and UPR to accommodate increased protein synthesis. In c‐Myc‐activated cells, knockout of protein kinase RNA‐like ER kinase (PERK), a key regulator of UPR, caused cell death because PERK‐mediated autophagy was inhibited. Moreover, Myc‐overexpression induced UPR in Drosophila and PERK was required for an increased autophagy‐dependent cell growth.^13^ Hypoxia also activated PERK transcription which induced UPR‐dependent upregulation of autophagy.^14^ Thus, autophagy can protect tumor cells by mediating the c‐Myc–PERK‐UPR axis. Autophagy also interacts with the mTOR pathway in tumor cells. p62/SQSTM1 is a key adapter in autophagy, can activate mTORC1 by promoting its interaction with Rag GTPases and TRAF6, facilitating its recruitment to lysosomes and eventually modulating autophagy. The regulation of mTORC1 by p62 promoted cell proliferation in vitro and tumor growth in vivo.^15^ mTOR also regulated V‐ATPase, a critical component of the late endosome/lysosome, through a transcription factor EB (TFEB) in renal cancer.^16^ That is to say, there is a regulatory network linking an oncogenic transcription factor TFEB to mTORC1 and lysosomal biogenesis, which allows autophagy to act as an oncosuppressor or oncopromoter.

The autophagy‐lysosomal pathway is a genetically programmed process, and cellular autophagy signaling pathways and autophagy‐lysosomal regulators, including the transcription factors and their coregulators, interact with one another. To support proper cellular function, the autophagy‐lysosomal function must be tightly controlled in different levels, eg, the epigenetic regulation, transcriptional regulation and post‐transcriptional regulation. There is considerable evidence to show that post‐translational modifications affect the localization, activity, and protein‐protein interactions of autophagy‐related proteins.^9^ However, the regulation of autophagy‐related and lysosomal function‐related genes on the epigenetic and transcriptional levels remain largely unexplored. In a few studies, researchers observed that the induction of autophagy was often accompanied by increased transcripts of certain autophagy genes, such as ATG7,^17^ ATG9,^18^ ATG12,^19^ and ATG14.^20^ The transcription of these genes were downregulated in human islets under lipotoxic conditions, which impairs autophagy.^21^ These studies suggest an unnegligible role of the transcriptional regulation of autophagy‐lysosomal function in tumorigenesis.

## Transcriptional regulation of the autophagy‐lysosomal function in cancer

Autophagy is induced in tumor cells by stressful conditions including hypoxia, nutrients starvation and cancer therapeutics.^22^ Deficient angiogenesis reduces the blood supply to tumor cells, elevating metabolic stress in response to insufficient nutrients, growth factors and oxygen. Cancer treatment is also infliction of damage and stress on tumor cells sufficient to kill them. As a result, autophagy is induced and modulated in cancer cells to tolerate stress and treatment. During this process, the multistep process of autophagy‐lysosomal pathway is extensively regulated at several levels, including post‐translationally through the action of conserved longevity factors. More recently, transcriptional regulation has emerged as an important mechanism for autophagy modulation.

Autophagy usually occurs in tumor cells distal to blood vessels to support tumor cell survival in hypoxic regions.^22^ The hypoxia inducible transcription factor 1α (Hif‐1α) drives the transcription of hundreds of genes that promote erythropoiesis and angiogenesis and decrease mitochondrial biogenesis and respiration, thus diminishing deleterious effects caused by O2 deficiency. A set of autophagy genes activated by Hif‐1α participates in the adaptive stress‐response as an important functional group to promote angiogenesis and alter metabolism.^23^

Nutrient starvation can also induce autophagy through transcription regulation. TFEB, a member of the basic helix‐loop‐helix leucine‐zipper family of transcription factors, promotes the transcription of several lysosomal genes by directly binding to specific E‐box sites on their promoters. The gene network promoted by TFEB is called the coordinated lysosomal expression and regulation (CLEAR).^24^ TFEB is therefore a master gene for lysosomal biogenesis, the process of which drives the expression of both autophagy and lysosomal genes so as to link two distinct types of cellular metabolism that cooperate in the autophagy‐lysosomal pathway. Starvation regulates TFEB activity, which is controlled by MAPK1/ERK2 and mTOR‐mediated phosphorylation of specific serine residues. During starvation, the phosphorylation of TFEB mediated by mTORC1 on the lysosomal surface is inhibited, which promotes TFEB nuclear translocation. Thus, autophagy and lysosomal biogenesis can be transcriptionally regulated by a lysosome‐to‐nucleus signaling mechanism via mTOR and TFEB. ZKSCAN3 is a transcriptional repressor of a large set of more than 60 genes that regulate autophagy and lysosome biogenesis/function. Starvation has the opposite effect on ZKSACN3 than TFEB, resulting in coordinately regulated lysosomal biogenesis and autophagy.^25^ Other transcription factors regulate autophagy‐lysosomal functions under different nutrient starvation conditions. For example, Rph1 is a negative regulator of the transcription of several ATG genes, overexpressing Rph1 inhibits autophagy, while its phosphorylated form induces autophagy. Rim15 has been reported to mediate the phosphorylation of Rph1 under nitrogen starvation, leading to the induction of autophagy.^26^ In hepatocellular carcinoma (HCC), PSMD10/Gankyrin translocates into the nucleus and binds with nuclear HSF1 (heat shock transcription factor 1) and ATG7 promoter to activate ATG7 expression, leading to the upregulation of autophagy in the advanced stage of starvation.^27^ In addition to those already mentioned, there are many other transcription factors which regulate autophagy and play an important role in cancer progress or during cancer treatment. The transcription factors and documented approaches for targeting transcriptional regulators that function as cancer therapies are detailed in Table 1.

**Table 1:** Transcriptional regulation of autophagy in response to oncogenic stress

Transcription factor	Inducer (s)	Target gene (s)	Therapy approaches
ATF5	Oncogenic stress	MTOR	Targeted biologic therapies such as dnATF5 peptides are under study.
GATA4	Chemotherapy	BCL2, ATG5, ATG7, ATG12, BECN1, FOXO1	
HSF1	Chemotherapy	ATG7	Minnelide is expected to begin Phase II clinical trial for treating gastrointestinal malignancies.
IRF1	Chemotherapy	BCL2, BCL2L2	
NAC1	Chemotherapy	HMGB1	
NF‐κB	Chemotherapy	BCL2, BCL2A1, BCL2L1, BECN1	NF‐κB inhibitors such as thalidomide, arsenic trioxide and bortezomib are currently in clinical use for cancer treatment.
NRF2	Chemotherapy	SQSTM1	Nrf2 modulators such as DMF, omaveloxolone, oltipraz, ML385, have already been investigated in clinical trials.
p53	Oncogenic stress	AEN, BAX, BBC3, C12orf5, CDKN2A, DAPK1, DRAM1, IGFBP3, PRKAB1, PRKAB2, SESN1, SESN2, BCL2, BCL2L1, MCL1	Early clinical trials are ongoing evaluating the antimutant p53 agent, PRIMA‐1MET, and specific MDM2–p53 nutlin antagonists.
p63	Chemotherapy	ATG3, ATG4A, ATG5, ATG7, ATG9A, ATG10, BECN1, MAP1LC3, NOS2, ULK1	
p73	Chemotherapy	ATG5, ATG7, DRAM1, UVRAG	
STAT3	Oncogenic stress	ADM, ATG3, BCL2, BCL2L1, BNIP3, CCL2, CTSB, CTSL, CXCL2, GADD45B, ICAM1, JUNB, MCL1, NPC1, THBS1	Many inhibitor targeting STAT3 such as Napabucasin and AZD9150, have also shown promising antioncogenic effects.

Many cancer therapeuties induce autophagy because they cause metabolic stress (2‐deoxyglucose, angiogenesis inhibitors), cellular damage (cytotoxic chemotherapy), or mimic factor deprivation/starvation to block growth signaling pathways (targeted noncytotoxic, kinase inhibitors). In breast cancer, chemotherapeutic agents can induce cytoprotective autophagy through transcriptional regulation of ATG7 controlled by HSF1.^28^ In ovarian cancer, nucleus accumbens‐1 plays an important role in autophagy induced by the chemotherapeutic drug cisplatin, mediated by the high‐mobility group box 1.^29^ Cisplatin can also induce ataxia telangiectasia mutated‐dependent phosphorylation of tumor protein p63 isoform, (ΔNp63α) in squamous cell carcinoma (SCC) cells, leading to transcriptional regulation of autophagic pathway members and specific microRNAs (miRNAs), which modulates the protein levels of ATG5, ATG6/Beclin1, ATG10, ATG12, ATG16L1 and UVRAG.^30^ Therefore, many cancer therapeutics lead to drug resistance mediated by autophagy, which impairs the therapeutic effect. Modulation of autophagy through transcriptional regulation may be a therapeutic target to improve current cancer treatment.

Besides these specific stress‐induced transcription factors, classic tumor suppressor p53 is a multiple stress‐responsive transcription factor. Many p53 target genes stimulate autophagy by inhibiting molecular cascades converging on mTOR. In response to stress, p53 can activate the following encoding genes: β1 and β2 AMPK subunits, tuberous sclerosis 2 (TSC2), phosphatase and tensin homolog (PTEN), sestrin 1 and 2, and insulin‐like growth factor binding protein 3 (IGFBP3), all of which functionally antagonize the autophagy‐suppressive functions of mTOR. p53‐inducible pro‐apoptotic members of the BCL‐2 protein family, such as BAX, BAD and BBC3, can stimulate autophagy by preventing inhibitory interactions of Beclin 1 with BCL‐2 and BCL‐XL.^31^ Furthermore, the lysosomal protein DRAM1 (damage‐regulated autophagy modulator), apoptosis enhancing nuclease (AEN), and death‐associated protein kinase 1 (DAPK1) play a role in the pro‐autophagy function of p53.^32^

Although autophagy plays a similar role in tumor cells as it does in normal cells, tumor cells encounter more stressful conditions including nutrient starvation, hypoxia, chemotherapy, and radiotherapy; therefore, they are more dependent on autophagy. This could be exploited to develop new cancer therapies targeting autophagy. Tumor masses are heterogeneous in vessel distribution and nutrient supply. Autophagy occurs in tumor cells that reside in hypoxic regions or are resistant to radiation and/or chemotherapy. Autophagy promotes the survival of this important subpopulation of tumor cells, providing an attractive target for cancer therapy. Since transcriptional regulation has emerged as an important mechanism for autophagy modulation, there has been an increasing interest in the study of transcriptional regulation of the autophagy‐lysosomal process. The transcriptional network is an important part of nuclear regulation of the autophagy‐lysosomal pathway, and is increasingly being investigated. However, the complexity of the transcriptional regulation of autophagy‐lysosomal functions and pathways highly elevates the difficulty of research. Further investigations are required to clarify the precise mechanism(s) of transcriptional regulation of autophagy.

## Epigenetic regulation of adaptive autophagy‐lyosome system

**Figure 1:**
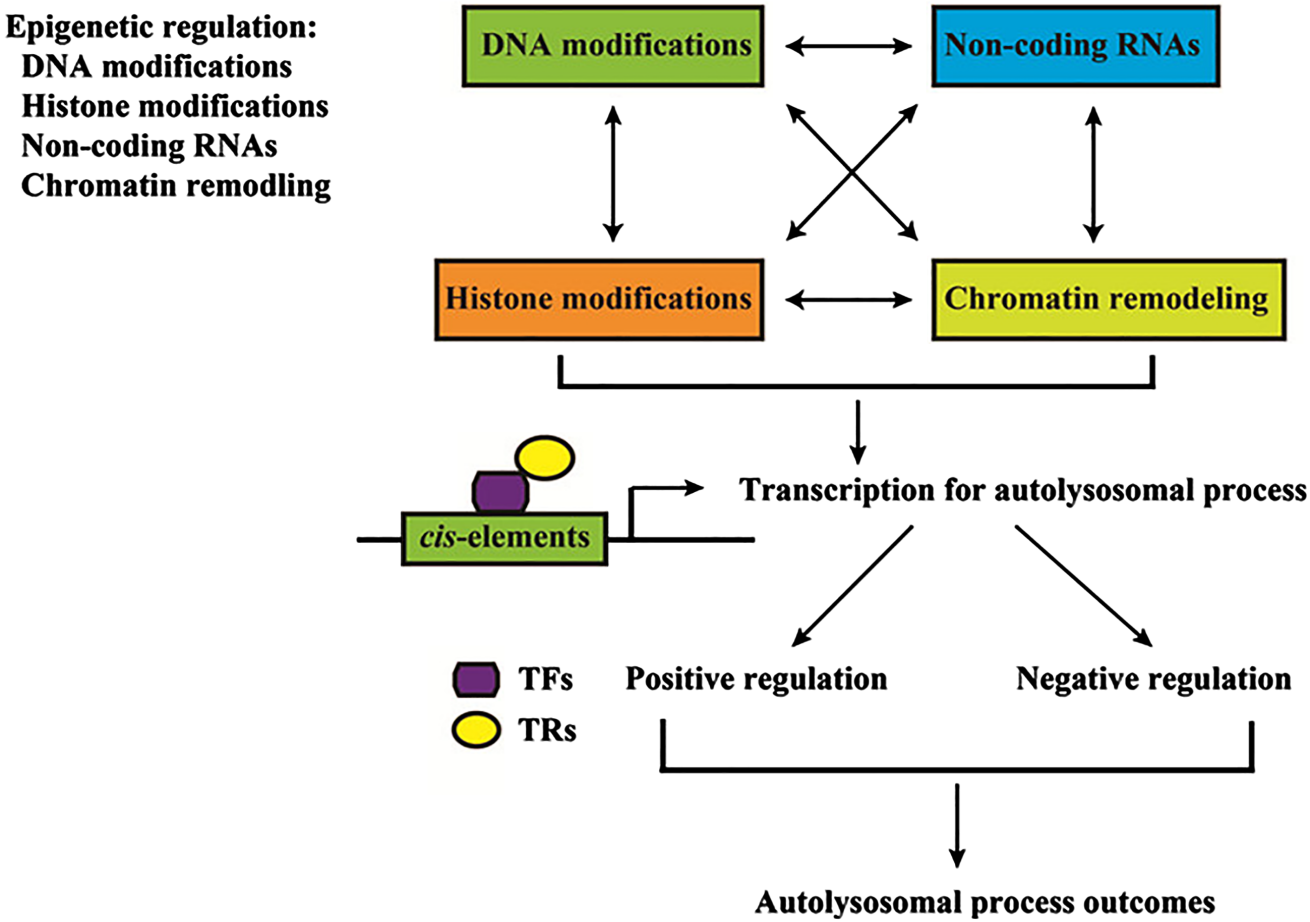
Transcriptional regulation of autophagy‐lysosomal pathway in cancer. Epigenetic abnormalities play an important role in transcriptional regulation and tumorigenesis. DNA and histone modifications, non‐coding RNAs, and chromatin remodeling interact to orchestrate biological outputs in favor of malignancy‐associated phenotypes, such as cellular differentiation, growth, invasion, metabolism, apoptosis, genomic instability and autophagy. Autophagy is modulated by transcription regulation by TF(s) and TR(s). Transcription of the autolysosomal process can be positive and negative, which determines the final outcomes.

Epigenetic abnormality plays an important role in gene transcriptional regulation and tumorigenesis. These modifications include DNA and histone modifications, non‐coding RNAs, and chromatin remodeling. These processes can interact and subsequently promote malignancy‐associated phenotypes, such as cellular differentiation, growth, invasion, metabolism, apoptosis, genomic instability, and modulation of autophagy (Fig 1).

Epigenetic alterations such as DNA and histone modifications, RNA interference, nucleosome positioning, and chromatin remodeling, can regulate the structure of chromatin and control gene expression,^33^ therefore interacting and subsequently promoting malignancy‐associated phenotypes, such as cellular differentiation, growth, invasion, metabolism, apoptosis, genomic instability, and modulation of autophagy (Fig 1). Therefore, the epigenetic regulation of autophagy‐lyosome system should be given more consideration as potential cancer treatments.

Histone modification and DNA methylation play crucial roles in controlling gene activity and nuclear architecture.^34^ Histone acetylation and deacetylation is a key molecular mechanism in autophagy. Histone acetylation is mediated by histone acetyltransferases (HATs) which activate gene transcription, whereas histone deacetylases (HDACs) suppress the transcription. When autophagy is induced, acetylation of histone H4 lysine 16 is reduced by the inhibition of the HAT hMOF (also called KAT8 or MYST1). In renal cell carcinoma cells, histone H3 acetylation prevents chronic mTOR inhibition, suggesting a positive role of HATs in the activation of autophagy.^35^ However, most HDACs are involved in autophagy in a nonepigenetic regulatory way. One of the HDACs, SIRT1, mainly influences autophagy through the deacetylation of key components including ATG5, ATG7, and ATG8.^36^ Histone methylation is also involved in the regulation of autophagy. G9a is a histone H3 lysine 9 (H3K9) methyltransferase, and it epigenetically represses genes that participate in autophagy through H3K9me2. Under normal conditions, G9a associates with the LC3B, WIPI1, and DOR gene promoters, epigenetically repressing them. Under starvation, however, G9a and G9a‐repressive histone marks are removed from these genes' promoters. The inhibition of G9a also induces autophagy and represses cell proliferation in neuroblastoma cells.^37^

Methylation of tumor suppressor gene promoters silences the gene expression and this has been reported in almost all cancer types. The inactivation of these genes also coordinates autophagy regulation. In non‐small cell lung cancer, cisplatin treatment inactivates sex determining region Y‐box 1 (SOX1) by hypermethylation of its promoter region, which enhances cisplatin‐mediated autophagy.^38^ In gastric cancer, Klotho gene promoter is frequently methylated, and Klotho expression promotes autophagy‐associated cell death.^39^ In malignant lymphoid tissues, the argininosuccinate synthetase‐1 promoter was hypermethylated to induce autophagy which enhanced the treatment of PEGylated arginine deiminase (ADI‐PEG20).^40^ Arginine methylation was recently shown to regulate the association of scaffold proteins with the cargo receptor complex to modulate selective autophagy.^41^ A single methionine can methylate PP2A, inhibiting autophagy.^42^

In addition, non‐coding RNAs regulate autophagy‐lysosome pathways. Accumulative evidence suggests that certain miRNAs genes participate in the regulation of autophagy. In SCC cells, miR‐885‐3p directly regulated ULK2 during autophagy induction upon cisplatin exposure.^43^ miR‐101 can sensitize breast cancer cells to 4‐hydroxytamoxifen (4‐OHT)‐mediated cell death by suppressing basal, etoposide‐ and rapamycin‐induced autophagy.^44^ miR‐204 inhibited LC3B‐mediated autophagy which suppressed renal clear cell carcinoma growth.^45^

Moreover, epigenetic alterations also play a key role in cancer with autophagy modulation and should be explored as potential cancer treatments. HDAC class IIb inhibitors (bufexamac) sensitized neuroblastoma cells to drug‐induced cell death by inhibiting autophagy.^46^ The HDAC inhibitors suberoylanilide hydroxamic acid (SAHA)/vorinostat and romidepsin have already been approved by FDA for the treatment of cutaneous T cell lymphoma. SAHA suppresses mTOR and activates the autophagic kinase ULK1 to stimulate autophagy.^47^ SAHA also triggers autophagy and enhances anticancer effects in breast cancer,^48^ ovarian cancer,^49^ hepatocellular carcinoma,^50^ and glioblastoma.^51^ Sirtinol is a class III HDAC inhibitor and induces autophagy‐mediated cell death by downregulating SIRT1/2 expression in MCF‐7 human breast cancer cells.^52^ The HDAC inhibitor valproic acid sensitized B16F10 melanoma cells to cucurbitacin B treatment.^53^ In addition, isoliquiritigenin, a natural inhibitor of autophagy‐related miR‐25, blocked chemoresistance‐associated autophagy to reverse drug resistance.^54^

Taken together, these studies suggest that epigenetic regulation of autophagy may provide prospective therapeutic approaches for cancer treatment.

## Future perspective

From a therapeutic perspective, understanding whether, when, and how autophagy can be harnessed to kill cancer cells remains challenging.^55^ Elucidating the balance of survival and death in the adaptive autophagy‐lysosomal pathways may improve cancer therapy and the treatment of metastasis. Investigating autophagy‐lysosomal mechanisms in cancers will help uncover how autophagy can be targeted as an alternative therapeutic strategy. Autophagy inhibitors have already been used to promote cell survival and autophagy inducers to promote cell death in various diseases, including cancer. Although the 1500 clinical trials using the mTOR inhibitor are ongoing (http://www.clinicaltrials.gov), the following questions regarding the signaling and transcriptional networks regulating autophagy‐lysosomal function still remain unanswered. Which epigenetic modifications represent critical molecular mechanisms of autophagy‐lysosomal regulation? How are the related transcription factors and transcriptional regulators involved in autophagy‐lysosomal regulation? Is this regulation reversible or irreversible? What is the consequence of this regulation in cancer cells? Can we predict the outcome of these modifications? Answers to these questions will help develop more personalized cancer treatment in the future. Understanding the mechanism behind autophagy‐lysosomal regulation and manipulating this regulation may contribute to novel strategies of oncosuppression.

## Disclosure

No authors report any conflict of interest.
